# Treatment of Ozæna

**Published:** 1881-09

**Authors:** 


TREATMENT OF OZÆNA.—M. Terullon writes on this subject, in the *Bu'l. Gen. de Therapeutique*, April: True ozæna, he says, includes those cases in which there is no osteal or periosteal leasion; in it the mucous membrane, when denuded of adherent crusts, is found to be almost intact, only slightly reddened, scarcely inflamed, thinned, and less vascular or velvety than usual. This thinning of the membrane is characteristc, and can be detected by the probe, which seems to come almost into direct contact with the bone. The nose is often blocked by greenish, fetid crusts, which the patient has great difficulty in expelling by blowing. These crusts may reach down to the pharynx and press on the soft palate. The patient sleeps with the mouth open, suffers constantly from headache, nausea, distate for food, and prolonged malaise. The manner in which this condition is developed is revealed on making a rhinoscopic examination. The nasal cavities are invariably found to be enormously dilated, forming a veritable antrum with no central constriction; the septum is visible along its whole extent, and sometimes also a portion of the posterior pharyngeal wall and the pharyngeal orifice of the Eustachian tube—a state of matters very different from that which characterizes the normal condition. The lower turbinated bone is usually also rudimentry. These features suggested to Zaufal the theory which is now very generally accepted regarding ozæna. According to him, inspiration takes little part in the removal of mucus from the nose; expiration, on the contrary, sweeps it horizontally forward to a more sensitive portion of the mucous membrane, where its presence excites the desire to clear the nose of obstruction. The inferior turbinated bone, besides narrowing the passage, accelerates and directs the current of air; when these bones are small and rudimentary, and the nasal cavities abnormally wide, the current of air has not sufficient force to carry the mucous secretion forward, so that the discharge accumulates, dries into crusts, and decomposes—whence the ordor described. So long as the accumulation is present the odor persists; when that is cleared away the odor disappears for a time. Zaufal's views are confirmed by what happens after the removal of large nasal polypi, when it is not unusual to have slight ozæna for a time, till the nares resume their normal volume.

The method of treatment which M. Terullon advocates, and describes as very successful, is based on the foregoing theory of origin of the affection. It consists first of thorough irrigation of the nasal cavities, with a weak solution of common salt, once every two or three days, or as often as twice a day, if necessary; and secondly, the insertion of a plug of cotton wool into each nostril, in such a way as to give the nares somewhat their normal conformation internally, and thus to remove the cause of the decomposition of the mucus. The cotton should be wrapped round the end of a knitting needle, so as to form a plug the length of the nasal fossa, *i. e.*, about 5 6 centimeters long; it should not be thicker than a penholder. The plug is then passed into the nose, being directed along its outer wall, pointing towards the outer angle of the eye, and thus taking the place of the inferior terbinated bone. When the plug is in place the needle is easily withdrawn. The plug should be renewed every two or three days, and is easily expelled by irrigation. The patient soon learns to apply it himself. Soon after, the mucus which comes from the nose will be found to be liquid, not fetid, and in its usual normal condition. This method of treatment, so long as it is faithfully carried out, keeps the affection effectually in check; but if it be neglected, even for a short time, the ozæna returns. As a rule, patients will not object to the trouble which it involves, as the maneuver is simple and easy, and the infirmity from which it relieves them is a very disagreeable one, both to themselves and their neighbors.

